# Performance of Large Language Models in Answering Healthcare Delivery Questions: A Quantitative Cross‐Sectional Study

**DOI:** 10.1002/hsr2.72634

**Published:** 2026-06-10

**Authors:** Mohsen Khosravi, Zahra Zamaninasab, Fatemeh Khosravi, Melika Attar, Morteza Arab‐Zozani

**Affiliations:** ^1^ Social Determinants of Health Research Center Birjand University of Medical Sciences Birjand Iran; ^2^ Department of Epidemiology and Biostatistics, School of Health, Social Determinants of Health Research Center Birjand University of Medical Sciences Birjand Iran; ^3^ School of Medicine Guilan University of Medical Sciences Rasht Iran; ^4^ School of Rehabilitation Sciences Babol University of Medical Sciences Babol Iran

**Keywords:** artificial intelligence, delivery of health care, education, generative artificial intelligence, large language models

## Abstract

**Background and Aims:**

The use of Large Language Model (LLM)‐based chatbots across various fields has yielded positive outcomes. Understanding the health service delivery system offers numerous benefits. This study aimed to analyze the performance of LLMs in answering healthcare delivery questions.

**Methods:**

A validated questionnaire relevant to the research context was administered to a sample of LLM‐based chatbots. The chatbots evaluated in this study included GPT‐4.1‐mini, Gemini 2.5, Copilot 2025, and Perplexity. A written prompt was provided to facilitate response generation by the chatbots. To analyze and compare the performance of the AI models in addressing the research questions, confusion matrices were constructed, and key metrics—sensitivity, specificity, positive predictive value, negative predictive value, and overall accuracy—were calculated.

**Results:**

The initial assessment of the chatbots showed perfect sensitivity (1.00), accurately identifying all true positives without false negatives. Specificity varied, with ChatGPT and Perplexity at 0.50, Gemini at 0.43, and Copilot at 0.33. Positive predictive values (PPV) ranged from 0.67 (Gemini) to 0.75 (ChatGPT and Perplexity), while negative predictive values (NPV) were uniformly perfect (1.00). Overall accuracy was highest for ChatGPT and Perplexity (0.80), with Gemini and Copilot at 0.73. In the second round, sensitivity remained perfect for all chatbots. Gemini achieved the highest specificity (0.80), followed by ChatGPT (0.67), Perplexity (0.60), and Copilot (0.50). PPVs improved, ranging from 0.75 (Copilot) to 0.91 (Gemini). NPVs remained perfect (1.00) across all models. Overall accuracy led by Gemini (0.93), with ChatGPT and Perplexity both at 0.87, and Copilot at 0.80.

**Conclusion:**

ChatGPT and Perplexity showed the highest initial performance, while the second round revealed improvements in most chatbots, especially in specificity and accuracy, with Gemini performing best. Further research is needed for deeper insights.

## 1 Introduction

Artificial Intelligence (AI) refers to the use of computer systems and technologies to simulate intelligent behavior and critical thinking similar to that of humans. The term was first introduced by John McCarthy in 1956, who described it as the science and engineering of creating intelligent machines [1]. An AI conversational chatbot is a computer program that is primarily established on a large language model (LLM), which utilizes natural language processing (NLP) to simulate conversations with users. These programs are capable of understanding and responding to user inquiries in a conversational manner. LLM‐based Chatbots employ machine learning and AI technologies to process information and generate natural, contextually appropriate responses [2, 3, 4, 5, 6].

NLP has progressed rapidly with the advent of transformer‐based architectures, including BERT and GPT [7, 8, 9]. These models capture complex linguistic structures and produce human‐like text, driving a transition from rule‐based methods to generative conversational systems. LLMs trained on extensive general and domain‐specific corpora now exhibit strong performance across diverse language tasks with minimal supervision [10]. LLM‐based chatbots hold considerable promise in clinical environments; however, their safe implementation requires rigorous evaluation comparable to that applied to new medical devices or pharmaceuticals [11, 12].

The research literature identifies several LLMs serving as chatbots that are utilized across various fields. ChatGPT, developed by OpenAI, has recently gained significant attention and has been evaluated in multiple studies as a tool for answering medical questions and providing high‐quality, empathetic responses [5, 13]. Google Bard, created by Google, has also been compared to other chatbots in certain studies [5, 14]. Bing AI, an artificial intelligence chatbot developed by Microsoft, is employed as a tool for answering questions and extracting data. This chatbot leverages advanced language models and, in some studies, has demonstrated superior performance in providing references and extracting information compared to other chatbots [15, 16]. Additionally, the Claude chatbot has been assessed in several studies within the field of dentistry, where it has shown acceptable accuracy and consistency in responding to dental‐related questions [14].

The use of LLM‐based chatbots across various fields has yielded positive outcomes. In the domain of women's health, chatbots have been effective in reducing anxiety and depression, as well as improving healthy relationships and self‐care behaviors among cancer patients [17]. Additionally, chatbots have facilitated access to cancer‐related information by simplifying complex content and helping to reduce healthcare disparities [18]. Compared to physicians, chatbots have been shown to provide more empathetic and high‐quality responses to patients' inquiries [13].

Furthermore, they have demonstrated effectiveness in promoting behavioral changes related to health, such as encouraging healthy lifestyles, smoking cessation, and improving treatment adherence. Chatbots also serve as a nonjudgmental platform for exchanging sensitive information [19].

The LLM‐based chatbots have demonstrated significant capabilities in answering questions across various domains, particularly in healthcare. Studies have presented that chatbots such as ChatGPT, GPT‐4, Claude, and Bing can generate responses of high‐quality, empathy, and readability, which in some cases have been rated superior to those provided by physicians. For instance, in a study comparing chatbot and physician responses to patient questions on social media, chatbot replies were preferred in 78.6% of cases and were rated significantly higher in terms of empathy and quality [13, 20]. Additionally, GPT‐4 has exhibited superior performance compared to other chatbots in addressing controversial topics within oral medicine and pathology [21]. However, limitations such as the generation of fabricated citations and occasional inaccuracies remain challenges for artificial intelligence chatbots [20, 21].

The health service delivery system is defined as the organization of individuals, institutions, and resources to provide health services to a target population. This system encompasses the provision of healthcare within a specific organizational setting, resulting in the implementation of various interventions [22, 23]. Focusing on quality, safety, efficiency, accessibility, and equity of health services, the system includes multiple subsystems such as public health, primary care, and specialized care, each playing a critical role in service delivery [23].

Understanding the health service delivery system offers numerous benefits. It serves as a comprehensive approach for designing, delivering, and improving health services, ultimately leading to enhanced patient outcomes [24]. Employing a systematic approach in the design and provision of health services reduces complication rates and improves service quality [23]. Additionally, awareness of this system aids in evaluating health service performance, including assessments of quality, safety, efficiency, accessibility, and equity [23]. The system also addresses the relationship between patient satisfaction, service quality, and physician–patient communication, thereby contributing to an improved patient experience [25]. Ultimately, knowledge of the health service delivery system facilitates the evolution of services by promoting coordination among various providers and levels of care, resulting in more effective and efficient healthcare delivery [23].

This study aimed to evaluate the performance of multiple LLMs in answering healthcare delivery questions. Despite numerous studies examining the performance of LLMs in healthcare, the reporting of certain valuable metrics—such as specificity, which reflects an LLM's ability to correctly identify false answers—has often been overlooked [26]. Furthermore, only a limited number of studies have employed real‐world data [27]. These gaps highlight the substantial value of research that reports underrepresented metrics like specificity while utilizing real‐world data, as demonstrated in the present study.

Evaluating the performance of LLM‐based chatbots in responding to questions related to the health service delivery system can offer numerous benefits to the research stakeholders. These stakeholders include policymakers, administrators, experts, and researchers within the health system, as well as developers of chatbots. By obtaining the research findings, they can identify strengths and weaknesses, recognize the best‐performing chatbots, and thereby facilitate improvements in both health service delivery and the functionality of the chatbots.

## 2 Methods

This study was a quantitative, cross‐sectional investigation conducted in the year 2025. The data from this study were reported in accordance with the STROBE guidelines for cross‐sectional studies [28].

### 2.1 Research Questions

The research's main question was formulated as follows: “What is the performance of LLMs in answering healthcare delivery questions?” For such a purpose, the following questions were articulated:

- What are the counts of true positive, false positive, true negative, and false negative responses generated by the chatbots in answering the research questions?
- What is the accuracy level of the chatbots in responding to the research questions?

### 2.2 Study Sample

In this study, a validated questionnaire relevant to the research context was administered to a sample of LLM‐based chatbots. The characteristics of both the questionnaire and the chatbots utilized are presented below.

### 2.3 Chatbots

The LLM‐based chatbots examined in this study included GPT‐4.1‐mini, Gemini 2.5, Copilot 2025, and Perplexity. These chatbots were selected due to their free availability on the World Wide Web, their international prominence—particularly as reflected in existing research literature as presented earlier in the introduction section—and their support for the English language.

### 2.4 Study Questionnaire

A pre‐designed, validated questionnaire available on the Study.com website, focusing on the domain of health system service delivery, was utilized. This test comprised 15 multiple‐choice questions in English. The rationale for selecting these questions was based on the international recognition of the website and the validation of the questionnaire's reliability and validity by the provider [29]. The options of the questionnaire were subsequently modified by the study authors to make them suitable for the analysis. This questionnaire encompassed a wide range of healthcare concepts, including public health and disparities such as unequal access to care among income groups, the role and benefits of long‐term care services for patients and families, and the distinctions between levels of healthcare providers such as primary, secondary, and tertiary care. It examined the scope of epidemiology in understanding disease distribution and causes, pharmacological safety through black box warnings, and the responsibilities of hospital‐based physicians. The items highlighted the pharmacist's role in patient access, the leadership duties of medical professionals in bridging staff and administration, and the challenges of evidence‐based practice, such as locating credible research. They emphasized the broad application of evidence‐based practice across healthcare, the importance of scientific research compilations, and the role of public health institutions such as the CDC. Furthermore, the questionnaire addressed patient privacy requirements under HIPAA, the definition and application of information technology in healthcare, and the greatest benefit of electronic medical records, which lies in improving the accessibility of patient information. The final version of the questionnaire is presented in Supporting Information S1: Appendix 1 (Study Questionnaire).

### 2.5 Data Gathering

In the initial phase of the study, the study questionnaire was presented to the chatbots described earlier. It is noteworthy that, to ensure the presence of true negative responses (i.e., genuinely incorrect answers) necessary for performing confusion matrix analysis on the provided answers, one of the response options for all questions was designated as “None.” Accordingly, the correct answer for one‐third of the questions (5 questions) was designed to be “None.” To blind the chatbots and reduce bias, this information was withheld from them.

To complete the response generation process by the chatbots, a written prompt was provided. To minimize bias and enhance the quality and accuracy of the results, this prompt was collaboratively drafted by two researchers and subsequently reviewed and approved by a third researcher. The prompt was as follows:

“Answer the Following Question Based on Your Knowledge.”

Due to the limitation on the number of words that could be input into the chatbots, it was not possible to present all research questions simultaneously. Therefore, each question was submitted separately, following a page refresh on the platform (to clear the chatbot's temporary memory and prepare it for optimal response generation).

To analyze the performance of the study chatbots with greater accuracy, precision, and detail, the prompt was presented to the chatbots multiple times over the course of 1 week. Initially, the prompt and corresponding questionnaire were provided to the chatbots, followed by the correct answers to facilitate the training process of the chatbots. After this 1‐week period, the questionnaire was administered again using the same prompt to evaluate the chatbots' performance after having acquired the correct answers.

Finally, the responses provided by the AI models were recorded in a data extraction table, including details such as the name of the AI model, the date of response, and the answer given. The gathered data were transcribed into a Word 2020 file. The entire data gathering process was conducted by one of the authors, specialized in working with LLM‐based chatbots. Finally, another researcher reviewed and verified the process to ensure data accuracy.

### 2.6 Data Analysis

After data collection, in alignment with the research literature, the definitive answers to each research question provided by the reference website were utilized as the gold standard (i.e., the definitive and correct answer for each question). These answers were subsequently reviewed and revalidated by three expert authors in the field of health service delivery, each holding a doctoral degree in Health Services Management, who served as the reference for assessing the accuracy of the responses generated by the AI models employed. To evaluate the answers provided by the AI models, one researcher compared the AI‐generated responses against the gold standard, categorizing them into two groups: “correct” and “incorrect”. At the conclusion of this process, another researcher reviewed and approved the procedure [30].

For the analysis and comparison of the performance of the AI models in answering the research questions, confusion matrices were constructed, and four metrics—sensitivity, specificity, positive predictive value, and negative predictive value—along with overall accuracy were calculated. The computations were performed by one researcher using Microsoft Excel 2016 and subsequently reviewed and validated by another researcher. The calculation methods for these metrics were as follows [30]:

- True Positive (TP): The number of correct AI responses among questions with a correct option other than “none” (i.e., selecting the correct option other than “none”).
- False Positive (FP): The number of incorrect AI responses through the selection of an incorrect option other than “none” (i.e., incorrectly selecting an option other than “none”).
- True Negative (TN): The number of correct AI responses among questions without a correct option other than “none” (i.e., correctly selecting “none”).
- False Negative (FN): The number of incorrect AI responses through incorrect selection of the “none” option (i.e., incorrectly selecting “none”).
- The sensitivity was calculated by dividing the number of true positives by the sum of true positives and false negatives:

(1) Sensitivity=TP/TP+FN

- Specificity was calculated by dividing the number of true negatives by the sum of true negatives and false positives:
(2) Specificity=TN/TN+FP
- Positive predictive value (PPV) was calculated by dividing the number of true positives by the sum of true positives and false positives:
(3) PPV=TP/TP+FP
- Negative predictive value (NPV) was calculated by dividing the number of true negatives by the sum of true negatives and false negatives:
(4) NPV=TN/TN+FN
- Finally, overall accuracy was determined by dividing the sum of true positives and true negatives by the total number of cases (true positives, true negatives, false positives, and false negatives):

(5) Accuracy=TP+TN/TP+TN+FP+FN

## 3 Results

The initial performance assessment of the chatbots in responding to the study questionnaire demonstrated that all of the four study chatbots—ChatGPT, Gemini, Copilot, and Perplexity—attained a sensitivity of 1.00, indicating flawless identification of true positive cases without any false negatives. Nonetheless, their specificity values exhibited variation, with ChatGPT and Perplexity achieving a specificity of 0.50, reflecting a moderate capacity to correctly identify true negatives, whereas Gemini and Copilot recorded lower specificity levels of 0.43 and 0.33, respectively. Similarly, the positive predictive values (PPV) ranged from 0.67 for Gemini to 0.75 for both ChatGPT and Perplexity, denoting the proportion of positive responses that were accurate. The negative predictive values (NPV) were uniformly perfect at 1.00 for all models, indicating that all negative responses were correctly classified. In terms of overall accuracy, ChatGPT and Perplexity led with a score of 0.80, while Gemini and Copilot each achieved an accuracy of 0.73 (Table 1).

**Table 1:** First round performance of chatbots.

Chatbot	TP	FP	TN	FN	Sensitivity	Specificity	PPV	NPV	Accuracy
ChatGPT	9	3	3	0	1.00	0.50	0.75	1.00	0.80
Gemini	8	4	3	0	1.00	0.43	0.67	1.00	0.73
Copilot	9	4	2	0	1.00	0.33	0.69	1.00	0.73
Perplexity	9	3	3	0	1.00	0.50	0.75	1.00	0.80

The second‐round performance evaluation of the study chatbots in responding to the questionnaire demonstrated that all chatbots maintained perfect sensitivity (1.00), effectively identifying all true positive cases without any false negatives. Gemini achieved the highest specificity at 0.80, followed by ChatGPT with 0.67, Perplexity at 0.60, and Copilot at 0.50. PPVs ranged from 0.75 for Copilot to 0.91 for Gemini, indicating varying degrees of precision in positive predictions. NPV remained uniformly perfect at 1.00 across all models, signifying the absence of misclassification among negative cases. Overall accuracy scores demonstrated Gemini leading with 0.93, followed by ChatGPT and Perplexity, each at 0.87, and Copilot at 0.80.

**Figure 1:**
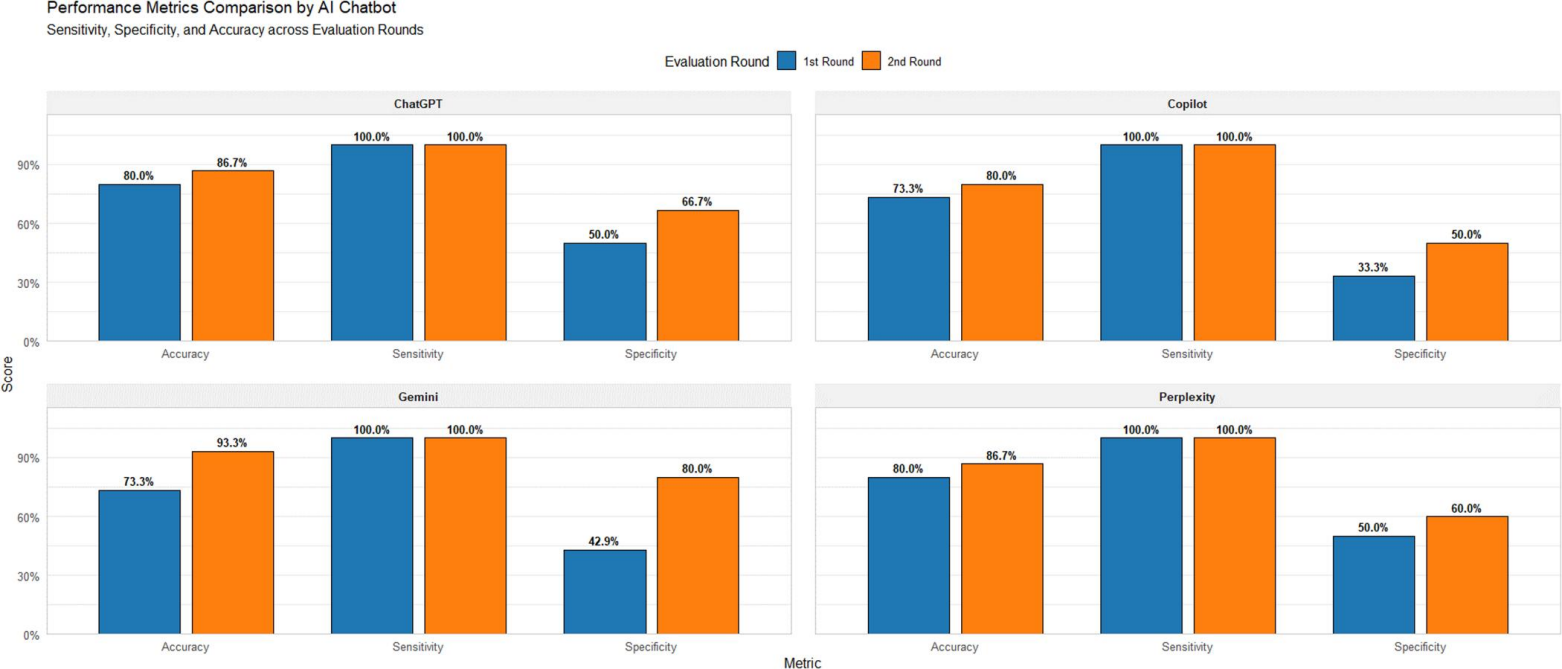
Accuracy, sensitivity, and specificity of LLMs.

Figure 1, presented herein, offers a comparative visualization of the performance metrics—sensitivity, specificity, and accuracy—for four leading chatbots (ChatGPT, Copilot, Gemini, and Perplexity) across two rounds of evaluation. This figure serves as a succinct graphical summary, complementing the tabular results described in Tables 1 and 2, and elucidates the trends and nuances observed in the chatbots' diagnostic performance when responding to healthcare delivery questions.

**Table 2:** Second round performance of chatbots.

Chatbot	TP	FP	TN	FN	Sensitivity	Specificity	PPV	NPV	Accuracy
ChatGPT	9	2	4	0	1.00	0.67	0.82	1.00	0.87
Gemini	10	1	4	0	1.00	0.80	0.91	1.00	0.93
Copilot	9	3	3	0	1.00	0.50	0.75	1.00	0.80
Perplexity	10	2	3	0	1.00	0.60	0.83	1.00	0.87

In both evaluation rounds, the sensitivity scores for all chatbots consistently remained at 100%, signifying an impeccable ability to identify true positive cases; this finding aligns with tabular data and corroborates the robust diagnostic strengths of these models. Conversely, the plot distinctly illustrates the variability in specificity among the chatbots, especially in the initial assessment. Gemini and Copilot exhibited lower specificity (42.9% and 33.3%, respectively), while ChatGPT and Perplexity demonstrated moderately higher values (50.0%). The second round marks a pronounced improvement, particularly for Gemini, which achieved the highest specificity (80.0%), followed by ChatGPT (66.7%), Perplexity (60.0%), and Copilot (50.0%).

The accuracy metric presented in the plot further accentuates the developmental trajectory across evaluations. ChatGPT and Perplexity achieved the highest accuracy in the initial round (80.0%), a trend sustained or improved in the subsequent round (86.7%). Notably, Gemini registered the most substantial gain in accuracy, advancing from 73.3% in the first round to 93.3% in the second, reflecting its enhanced overall classification performance post‐training. Copilot exhibited incremental improvement but consistently lagged behind the other models in both rounds (Figure 1).

**Figure 2:**
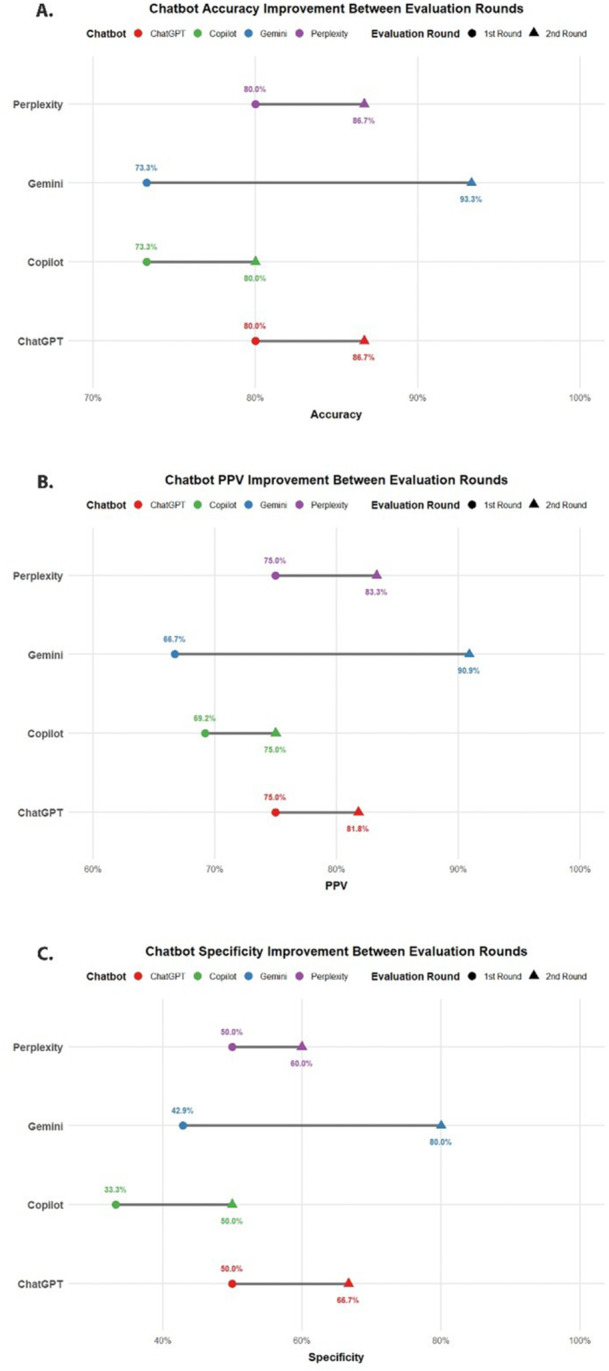
Performance improvement of LLMs. A (Overall Accuracy), B (Positive Predictive Value), and C (Specificity).

The performance trends of the four chatbots across the two evaluation rounds are further visualized in Figure 2, which includes panels A (overall accuracy), B (positive predictive value), and C (specificity). Figure 2A illustrates the improvement in overall accuracy from the first to the second round, with Gemini demonstrating the most substantial gain, rising from 73.3% to 93.3%, and subsequently achieving the highest final accuracy. ChatGPT and Perplexity both showed an improvement to 86.7%, while Copilot's accuracy increased modestly to 80.0%.

Similarly, Figure 2B depicts the enhancement in the chatbots' precision, as measured by the PPV. Gemini again showed the most remarkable improvement, with its PPV increasing from 66.7% to 90.9%, indicating a superior ability to minimize false positives in the second round. The PPV for Perplexity and ChatGPT also improved to 83.3% and 81.8%, respectively, while Copilot's PPV saw a more moderate increase to 75.0%.

The variability and improvement in the models' ability to correctly identify true negatives are presented in Figure 2C. The chart confirms the initial challenge all models faced with this metric in the first round. Gemini exhibited the most pronounced learning effect, with its specificity rising from 42.9% to 80.0%. ChatGPT also showed a strong improvement from 50.0% to 66.7%, while Perplexity and Copilot improved to 60.0% and 50.0%, respectively.

**Figure 3:**
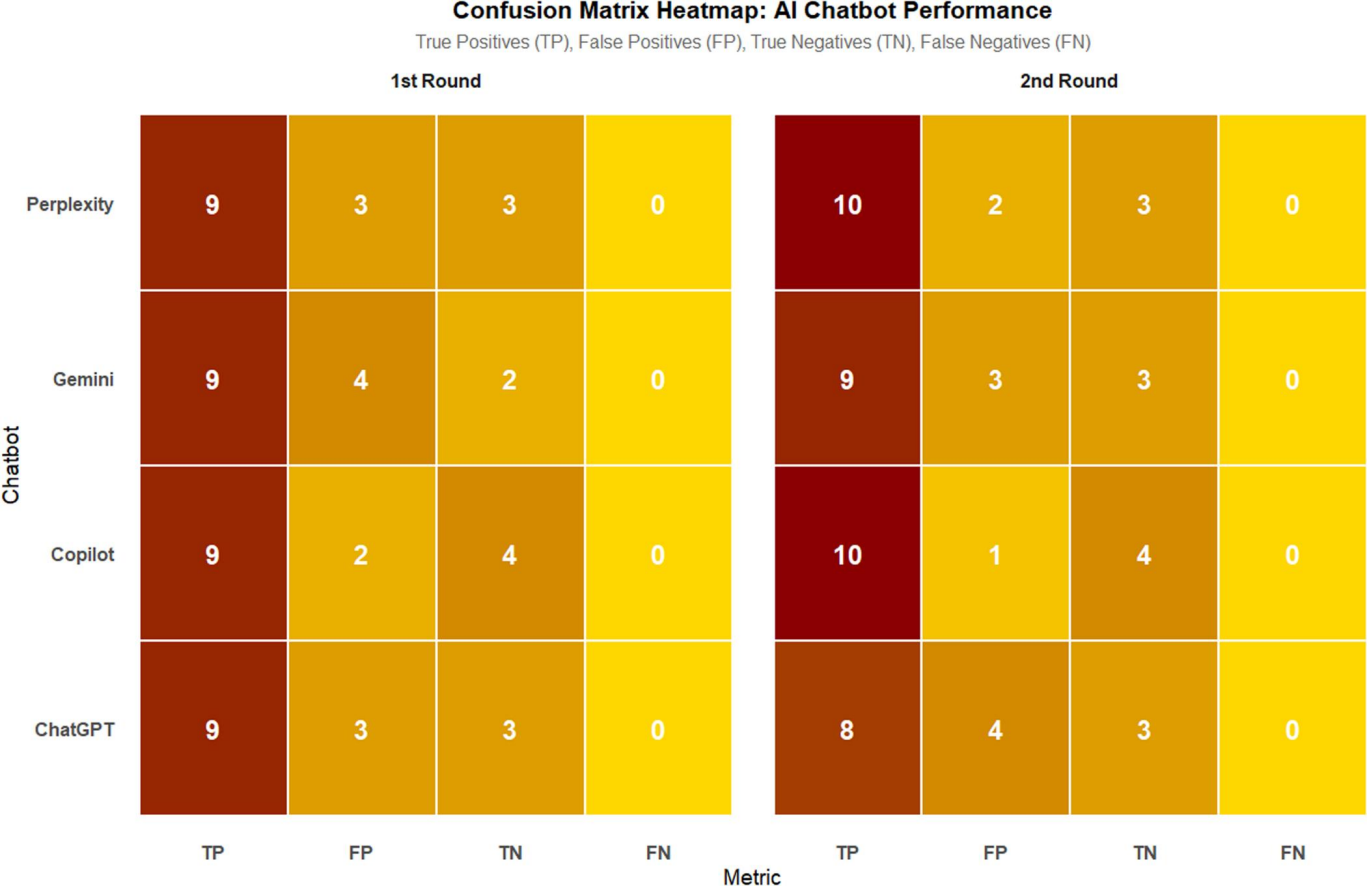
Heatmap visualization of the underlying confusion matrices for LLMs.

Figure 3 provides a consolidated heatmap visualization of the underlying confusion matrices for all chatbots across both evaluation rounds. This figure allows for a direct, comparative analysis of the raw counts of True Positives (TP), False Positives (FP), True Negatives (TN), and False Negatives (FN) that form the basis for the calculated metrics.

The heatmap immediately confirms the consistent absence of False Negatives (FN) across all models and both rounds, corresponding to the perfect sensitivity (1.00) reported in Tables 1 and 2. The primary performance variations are clearly attributed to differences in False Positives (FP) and True Negatives (TN). In the first round, higher FP counts (ranging from 3 to 4) and lower TN counts (ranging from 2 to 3) are evident for all models. The second round shows a visible improvement: a reduction in FP counts is observed for Gemini (from 4 to 1) and ChatGPT (from 3 to 2), alongside an increase in TN counts for ChatGPT (from 3 to 4) and Gemini (from 3 to 4). This graphical shift towards higher TN and lower FP values provides a clear visual explanation for the corresponding improvements in specificity and PPV documented in the other figures and tables.

The findings from the first round of responses indicated that the chatbots collectively failed to answer questionnaire items 1, 2, and 7. In addition, Copilot failed to answer item 10, while Gemini failed to answer item 15. Meanwhile, the findings from the second round of responses revealed that all chatbots failed to answer item 1, with the exception of Gemini, which succeeded. Furthermore, ChatGPT and Copilot failed to answer item 7, and both Copilot and Perplexity failed to answer item 11 of the questionnaire.

## 4 Discussion

The study results demonstrated notable trends in the performance of the evaluated chatbots—ChatGPT, Gemini, Copilot, and Perplexity—across two rounds of assessment in accurately responding to the study questionnaire. In the initial round, all chatbots exhibited perfect sensitivity (1.00), indicating flawless identification of true positive cases. However, variability was observed in specificity. ChatGPT and Perplexity each attained a specificity of 0.50, reflecting a moderate capability to reject false positives, whereas Gemini and Copilot recorded lower specificity values of 0.43 and 0.33, respectively, signifying a higher incidence of false positives. Correspondingly, PPV was highest for ChatGPT and Perplexity (0.75), with Gemini and Copilot performing less favorably. Accuracy scores mirrored these distinctions, with ChatGPT and Perplexity achieving 0.80, while Gemini and Copilot both scored 0.73.

The study results demonstrated that the performance of the LLMs in terms of specificity was lower compared to their performance on other metrics. In this regard, in line with our study findings, several studies have reported the comparatively lower performance of LLMs in terms of specificity relative to other metrics [31, 32, 33]. This finding may hold significant implications for the stakeholders within the relevant context. Conversely, the apparently lower performance of Gemini aligns with the findings reported in previous studies in the literature [34, 35]. The perceived lower performance of Gemini has been attributed to the increased complexity of the multiple‐choice questions, particularly at advanced difficulty levels, where it demonstrated significantly reduced accuracy compared to easier question sets [34, 36, 37].

The second round of performance analysis revealed improvements across most chatbots, particularly in specificity and overall accuracy. In this context, the specificity of ChatGPT, Gemini, Copilot, and Perplexity improved by 17%, 37%, 17%, and 10%, respectively, while their overall accuracy increased by 7%, 20%, 7%, and 7%, respectively. ChatGPT increased its specificity from 0.50 to 0.67, accompanied by enhancements in PPV (0.82) and accuracy (0.87). Gemini demonstrated the most significant advancement, elevating its specificity to 0.80 and attaining the highest PPV (0.91) and accuracy (0.93) among all chatbots in this round. Perplexity also showed considerable improvement, with specificity rising to 0.60, PPV to 0.83, and accuracy to 0.87. Copilot, while maintaining perfect sensitivity, exhibited only a modest increase in specificity (0.50) and overall accuracy (0.80), indicating a slower rate of improvement relative to its counterparts. These results may have significant implications for the beneficiaries, as they indicate that Gemini possesses considerable potential as an LLM to be further trained and to achieve enhanced performance relative to other LLMs. This finding was consistent with previous literature, which highlighted Gemini's significant potential for training and its capacity to achieve higher performance [38].

The existing literature reports that scaling model size alongside effective training and instruction tuning significantly enhances comprehension, reasoning, knowledge recall, and factual accuracy in LLMs [39]. Additionally, few‐shot learning improves clinical named entity recognition and other specialized tasks, enabling high performance with minimal additional data [40]. Furthermore, training with synthetic data augmentation boosts downstream model accuracy, underscoring the importance of targeted training data in healthcare applications [41].

Overall, the performance of the studied chatbots across both rounds consistently presented sensitivity and NPV of 1.00. This consistency underscored their strong ability to correctly identify positive cases and reliably confirm true negative cases, highlighting the robustness of these models in minimizing false negatives. The findings suggested that, although all chatbots reliably captured true positives, enhancements in specificity and PPV were realized primarily in the second round, notably for Gemini and Perplexity, which contributed to superior accuracy. These improvements suggested enhanced discrimination between true and false positives following the chatbots' learning process, with Gemini exhibiting the most pronounced gains in specificity and accuracy, indicating it derived the greatest benefit. Conversely, Copilot, despite maintaining high sensitivity, demonstrated more limited progress in reducing false positives, adversely affecting its overall accuracy relative to the other chatbots. ChatGPT and Perplexity maintained a balanced performance between sensitivity and specificity across both rounds, resulting in consistently strong outcomes. In this context, the findings of our study offer valuable insights for stakeholders aiming to enhance the performance of LLMs through training.

As indicated by the findings of our study, in the initial round of responses, the chatbots collectively failed to answer questionnaire items 1, 2, and 7. An examination of the questionnaire revealed that items 1 and 2 were both questions requiring a true negative response. In contrast, item 7 was an analytical, qualitative question concerning the process through which patients accessed pharmacists during the course of healthcare delivery. The initial performance of the chatbots on items 1 and 2 was consistent with the earlier findings concerning questions requiring a true negative response, as discussed in detail in the preceding section of this study [31, 32, 33]. Meanwhile, the inability of the chatbots to correctly address the analytical context of questionnaire item 7 was consistent with findings in the literature, which indicate that chatbots frequently encounter difficulties when confronted with complex analytical contexts [42, 43, 44]. Meanwhile, when considering the performance of the chatbots in the second round, ChatGPT and Gemini demonstrated their strength through training by successfully answering item 7, in contrast to Copilot and Perplexity, which failed to provide correct responses.

While this study highlighted the notably strong performance of LLM‐based chatbots in healthcare, the ethical challenges associated with deploying such technologies in clinical systems must not be overlooked. In this regard, the integration of LLM‐based chatbots into healthcare services presents several ethical challenges, including output bias, privacy concerns, regulatory compliance, transparency, and the risk of human role displacement [45, 46, 47]. Meanwhile, incorporating human‐centered values into these technologies has been identified as a key strategy for addressing such ethical issues [48].

## 5 Limitations and Implications

This study had a limitation. Given the rapid pace of chatbot development and the frequent release of new versions, the versions employed in this research may not correspond to the most current ones at the time of publication. Future studies using similar methodologies could address this limitation by evaluating newer versions of the chatbots. This study also had several implications to address. In this regard, the study reported the performance of several LLM‐based chatbots to health system stakeholders to facilitate planning for the implementation of such chatbots within the health system. The study also presented the strengths and weaknesses of these chatbots to their developers, which facilitates planning aimed at improving the performance and quality of the chatbots. In this context, the study revealed that LLMs exhibit lower specificity compared to other performance metrics. This finding has important implications for beneficiaries, particularly those intending to use LLMs in clinical domains such as diagnostic processes, where confirming the presence or absence of a disease is critical. Chatbot developers can leverage this data to improve their models, while future researchers may utilize the findings to conduct similar investigations, thereby enriching the understanding of the phenomenon. Additionally, the study provides novel insights into LLM performance post‐training, offering valuable guidance for stakeholders seeking to enhance LLM capabilities through training. Further research is encouraged to deepen insights into this subject.

## 6 Conclusion

The study results revealed significant trends in the performance of the evaluated chatbots—ChatGPT, Gemini, Copilot, and Perplexity—across two rounds of assessment in accurately responding to the study questionnaire. While ChatGPT and Perplexity demonstrated the highest levels of performance in the initial round, the second round of analysis showed improvements across most chatbots, particularly in specificity and overall accuracy, with Gemini exhibiting the highest performance among them. Further research is warranted to provide deeper insights into this subject.

## Author Contributions

**Mohsen Khosravi:** conceptualization, formal analysis, writing – original draft, project administration, investigation, and methodology. **Zahra Zamaninasab:** writing – review and editing, resources, formal analysis, visualization, and validation. **Fatemeh Khosravi:** resources. **Melika Attar:** resources and investigation. **Morteza Arab‐Zozani:** validation, conceptualization, project administration, formal analysis, supervision, writing – review and editing.

## Ethics Statement

The research was approved by Birjand University of Medical Sciences (BUMS) ethical committee in Iran with the ethical code: IR.BUMS.REC.1404.149. Throughout all phases of this research, all ethical principles related to conduction of the research were strictly adhered to. Finally, data extracted from this research was to be retained by researchers for up to 3 years following the study publication.

## Conflicts of Interest

The authors declare no conflicts of interest.

## Transparency Statement

The first author, Mohsen Khosravi, affirms that this manuscript is an honest, accurate, and transparent account of the study being reported; that no important aspects of the study have been omitted; and that any discrepancies from the study as planned (and, if relevant, registered) have been explained.

## Supporting information

Supporting File 1

## Data Availability

The research data can be accessed by contacting the corresponding author of the paper.

## References

[1] Amisha , P. Malik , M. Pathania , and V. K. Rathaur , “Overview of Artificial Intelligence in Medicine,” Journal of Family Medicine and Primary Care 8 (2019): 2328–2331. 10.4103/jfmpc.jfmpc_440_19 PMC6691444 31463251

[2] C. Chakraborty , S. Pal , M. Bhattacharya , S. Dash , and S. S. Lee , “Overview of Chatbots With Special Emphasis on Artificial Intelligence‐Enabled ChatGPT in Medical Science,” Frontiers in Artificial Intelligence 6 (2023): 1237704. 38028668 10.3389/frai.2023.1237704 PMC10644239

[3] E. Adamopoulou and L. Moussiades , “An Overview of Chatbot Technology,” Artificial Intelligence Applications and Innovations 584 (2020): 373–383.

[4] B. Huo , A. Boyle , N. Marfo , et al., “Large Language Models for Chatbot Health Advice Studies: A Systematic Review,” JAMA Network Open 8 (2025): e2457879. 39903463 10.1001/jamanetworkopen.2024.57879 PMC11795331

[5] T. P. Nguyen , B. Carvalho , H. Sukhdeo , et al., “Comparison of Artificial Intelligence Large Language Model Chatbots in Answering Frequently Asked Questions in Anaesthesia,” BJA Open 10 (2024): 100280. 38764485 10.1016/j.bjao.2024.100280 PMC11099318

[6] H. S. Yang , F. Wang , M. B. Greenblatt , S. X. Huang , and Y. Zhang , “AI Chatbots in Clinical Laboratory Medicine: Foundations and Trends,” Clinical Chemistry 69 (2023): 1238–1246. 37664912 10.1093/clinchem/hvad106

[7] J. C. L. Chow , V. Wong , and K. Li , “Generative Pre‐Trained Transformer‐Empowered Healthcare Conversations: Current Trends, Challenges, and Future Directions in Large Language Model‐Enabled Medical Chatbots,” BioMedInformatics 4 (2024a): 837–852.

[8] S. Locke , A. Bashall , S. Al‐Adely , J. Moore , A. Wilson , and G. B. Kitchen , “Natural Language Processing in Medicine: A Review,” Trends in Anaesthesia and Critical Care 38 (2021): 4–9.

[9] A. Babu and S. B. Boddu , “Bert‐Based Medical Chatbot: Enhancing Healthcare Communication Through Natural Language Understanding,” Exploratory Research in Clinical and Social Pharmacy 13 (2024): 100419. 38495953 10.1016/j.rcsop.2024.100419 PMC10940906

[10] J. C. L. Chow and K. Li , “Large Language Models in Medical Chatbots: Opportunities, Challenges, and the Need to Address AI Risks,” Information [Online] 16, no. 7 (2025): 549.

[11] J.‐E. Bibault , B. Chaix , A. Guillemassé , et al., “A Chatbot Versus Physicians to Provide Information for Patients With Breast Cancer: Blind, Randomized Controlled Noninferiority Trial,” Journal of Medical Internet Research 21 (2019): e15787. 31774408 10.2196/15787 PMC6906616

[12] S. Siddique and J. C. L. Chow , “Machine Learning in Healthcare Communication,” Encyclopedia 1 (2021): 220–239.

[13] J. W. Ayers , A. Poliak , M. Dredze , et al., “Comparing Physician and Artificial Intelligence Chatbot Responses to Patient Questions Posted to a Public Social Media Forum,” JAMA Internal Medicine 183 (2023): 589–596. 37115527 10.1001/jamainternmed.2023.1838 PMC10148230

[14] R. Rokhshad , M. Fadul , G. Zhai , K. Carr , J. G. Jackson , and P. Zhang , “A Comparative Analysis of Responses of Artificial Intelligence Chatbots in Special Needs Dentistry,” Pediatric Dentistry 46 (2024): 337–344. 39420494

[15] J. E. Hill , C. Harris , and A. Clegg , “Methods for Using Bing's AI‐Powered Search Engine for Data Extraction for a Systematic Review,” Research Synthesis Methods 15 (2024): 347–353. 38066713 10.1002/jrsm.1689

[16] A. K. Mcmahon , R. S. Terry , W. E. Ito , W. R. Molina , and B. B. Whiles , “Battle of the Bots: A Comparative Analysis of ChatGPT and Bing AI for Kidney Stone‐Related Questions,” World Journal of Urology 42 (2024): 600. 39470812 10.1007/s00345-024-05326-1

[17] H. K. Kim , “The Effects of Artificial Intelligence Chatbots on Women's Health: A Systematic Review and Meta‐Analysis,” Healthcare 12 (2024): 534. 38470645 10.3390/healthcare12050534 PMC10930454

[18] A. A. Abreu , G. Z. Murimwa , E. Farah , et al., “Enhancing Readability of Online Patient‐Facing Content: The Role of AI Chatbots in Improving Cancer Information Accessibility,” Journal of the National Comprehensive Cancer Network 22, no. 2D (2024): e237334, 10.6004/jnccn.2023.7334. 38749478

[19] A. Aggarwal , C. C. Tam , D. Wu , X. Li , and S. Qiao , “Artificial Intelligence‐Based Chatbots for Promoting Health Behavioral Changes: Systematic Review,” Journal of Medical Internet Research 25 (2023): e40789. 36826990 10.2196/40789 PMC10007007

[20] D. Chen , R. Parsa , A. Hope , et al., “Physician and Artificial Intelligence Chatbot Responses to Cancer Questions From Social Media,” JAMA Oncology 10 (2024): 956–960. 38753317 10.1001/jamaoncol.2024.0836 PMC11099835

[21] H. Mohammad‐Rahimi , Z. H. Khoury , M. I. Alamdari , et al., “Performance of AI Chatbots on Controversial Topics in Oral Medicine, Pathology, and Radiology,” Oral Surgery, Oral Medicine, Oral Pathology and Oral Radiology 137 (2024): 508–514. 38553304 10.1016/j.oooo.2024.01.015

[22] G. Fanjiang , J. H. Grossman , W. D. Compton , and P. P. Reid , “National Academy of Engineering (US) and Institute of Medicine (US) Committee on Engineering and the Health Care System.” in Building a Better Delivery System: A New Engineering/Health Care Partnership, ed. P. P. Reid , w. D. Compton , J. H. Grossman , and G. Fanjiang (National Academies Press (US), 2005). 20669457

[23] I. Papanicolas , D. Rajan , M. Karanikolos , A. Soucat , and J. F. Marimont , eds., Health System Performance Assessment: A Framework For Policy Analysis, (European Observatory on Health Systems and Policies, 2022). 37023239

[24] A. Komashie , J. Ward , T. Bashford , et al., “Systems Approach to Health Service Design, Delivery and Improvement: A Systematic Review and Meta‐Analysis,” BMJ Open 11 (2021): e037667. 10.1136/bmjopen-2020-037667 PMC7817809 33468455

[25] M. Cowing , C. M. Davino‐Ramaya , K. Ramaya , and J. Szmerekovsky , “Health Care Delivery Performance: Service, Outcomes, and Resource Stewardship,” Permanente Journal 13 (2009): 72–78. 20740107 10.7812/tpp/08-100 PMC2911834

[26] E. J. Gong , C. S. Bang , J. J. Lee , and G. H. Baik , “Knowledge‐Practice Performance Gap in Clinical Large Language Models: Systematic Review of 39 Benchmarks,” Journal of Medical Internet Research 27 (2025): e84120. 41325597 10.2196/84120 PMC12706444

[27] P. S. Suh , S. Y. Jeong , D. Ueda , et al., “Insufficient Reporting Quality in Large Language Model Studies in the Field of Radiology,” Insights into Imaging 17 (2026): 71. 41838317 10.1186/s13244-026-02236-1 PMC12992711

[28] E. Von Elm , D. G. Altman , M. Egger , S. J. Pocock , P. C. Gøtzsche , and J. P. Vandenbroucke , “The Strengthening the Reporting of Observational Studies in Epidemiology (STROBE) Statement: Guidelines for Reporting Observational Studies,” Annals of Internal Medicine 147 (2007): 573–577. 17938396 10.7326/0003-4819-147-8-200710160-00010

[29] Study.com , “Health 307: Healthcare Delivery Systems Final Exam [Online],” published 2025, https://study.com/academy/exam/course/health-307-healthcare-delivery-systems.html.

[30] R. Parikh , A. Mathai , S. Parikh , G. Chandra Sekhar , and R. Thomas , “Understanding and Using Sensitivity, Specificity and Predictive Values,” Indian Journal of Ophthalmology 56, no. 1 (2008): 45–50, 10.4103/0301-4738.37595. 18158403 PMC2636062

[31] F. Y. Al‐Ashwal , M. Zawiah , L. Gharaibeh , R. Abu‐Farha , and A. N. Bitar , “Evaluating the Sensitivity, Specificity, and Accuracy of ChatGPT‐3.5, ChatGPT‐4, Bing AI, and Bard Against Conventional Drug‐Drug Interactions Clinical Tools,” Drug, Healthcare and Patient Safety 15 (2023): 137–147. 37750052 10.2147/DHPS.S425858 PMC10518176

[32] C. Y. K. Williams , B. Y. Miao , A. E. Kornblith , and A. J. Butte , “Evaluating the Use of Large Language Models to Provide Clinical Recommendations in the Emergency Department,” Nature Communications 15 (2024): 8236. 10.1038/s41467-024-52415-1 PMC11461960 39379357

[33] D. li , L. Wu , M. Zhang , et al., “Assessing the Performance of Large Language Models in Literature Screening for Pharmacovigilance: A Comparative Study,” Frontiers in Drug Safety and Regulation 4 (2024): 2024. 10.3389/fdsfr.2024.1379260 PMC12443093 40979379

[34] I. M. Salman , O. Z. Ameer , M. A. Khanfar , and Y. H. Hsieh , “Artificial Intelligence in Healthcare Education: Evaluating the Accuracy of ChatGPT, Copilot, and Google Gemini in Cardiovascular Pharmacology,” Frontiers in Medicine 12 (2025): 1495378. 40046930 10.3389/fmed.2025.1495378 PMC11879995

[35] I. Aksoy and M. Kara Arslan , “Comparison of Performance of Artificial Intelligence Tools in Answering Emergency Medicine Question Pool: ChatGPT 4.0, Google Gemini and Microsoft Copilot,” Pakistan Journal of Medical Sciences 41 (2025): 968–972. 40290213 10.12669/pjms.41.4.11178 PMC12022595

[36] C. A. Gomez‐Cabello , S. Borna , S. M. Pressman , S. A. Haider , and A. J. Forte , “Large Language Models for Intraoperative Decision Support in Plastic Surgery: A Comparison Between ChatGPT‐4 and Gemini,” Medicina 60 (2024): 957. 38929573 10.3390/medicina60060957 PMC11205293

[37] S. Sau , D. D. George , R. Singh , et al., “Accuracy and Quality of ChatGPT‐4o and Google Gemini Performance on Image‐Based Neurosurgery Board Questions,” Neurosurgical Review 48 (2025): 320. 40131528 10.1007/s10143-025-03472-7

[38] G. Mondillo , V. Frattolillo , S. Colosimo , et al., “Basal Knowledge in the Field of Pediatric Nephrology and Its Enhancement Following Specific Training of ChatGPT‐4 “Omni” and Gemini 1.5 Flash,” Pediatric Nephrology 40 (2025): 151–157. 39150524 10.1007/s00467-024-06486-3 PMC11584465

[39] K. Singhal , S. Azizi , T. Tu , et al., “Large Language Models Encode Clinical Knowledge,” Nature 620 (2023): 172–180. 37438534 10.1038/s41586-023-06291-2 PMC10396962

[40] Y. Hu , Q. Chen , J. Du , et al., “Improving Large Language Models for Clinical Named Entity Recognition via Prompt Engineering,” Journal of the American Medical Informatics Association 31 (2024): 1812–1820. 38281112 10.1093/jamia/ocad259 PMC11339492

[41] C. R. Woolsey , P. Bisht , J. Rothman , and G. Leroy , “Utilizing Large Language Models to Generate Synthetic Data to Increase the Performance of BERT‐Based Neural Networks,” AMIA Joint Summits on Translational Science Proceedings 2024 (2024): 429–438. 38827067 PMC11141799

[42] V. Mavrych , E. M. Yousef , A. Yaqinuddin , and O. Bolgova , “Large Language Models in Medical Education: A Comparative Cross‐Platform Evaluation in Answering Histological Questions,” Medical Education Online 30 (2025): 2534065. 40651009 10.1080/10872981.2025.2534065 PMC12258195

[43] D. Wu , J. Yang , and K. Wang , “Exploring the Reversal Curse and Other Deductive Logical Reasoning in BERT and GPT‐Based Large Language Models,” Patterns 5 (2024): 101030. 39568650 10.1016/j.patter.2024.101030 PMC11573886

[44] R. Bhayana , S. Krishna , and R. R. Bleakney , “Performance of Chatgpt on a Radiology Board‐Style Examination: Insights into Current Strengths and Limitations,” Radiology 307 (2023): e230582. 37191485 10.1148/radiol.230582

[45] J. C. L. Chow , V. Wong , and K. Li , “Generative Pre‐Trained Transformer‐Empowered Healthcare Conversations: Current Trends, Challenges, and Future Directions in Large Language Model‐Enabled Medical Chatbots,” BioMedInformatics [Online] 4 (2024): 837–852, 10.3390/biomedinformatics4010047.

[46] M. Khosravi , Y. Herandi , S. S. Tabatabaei Far , et al., “Psychometric Properties of an Iranian Instrument for Assessing Adherence to Ethical Principles in the Use of Artificial Intelligence Among Healthcare Providers,” International Journal of Medical Informatics 203 (2025): 106019. 40582296 10.1016/j.ijmedinf.2025.106019

[47] M. Khosravi , Z. Zare , S. M. Mojtabaeian , and R. Izadi , “Ethical Challenges of Using Artificial Intelligence in Healthcare Delivery: A Thematic Analysis of a Systematic Review of Reviews,” Journal of Public Health 33 (2025b): 2485–2495.

[48] J. C. L. Chow and K. Li , “Ethical Considerations in Human‐Centered AI: Advancing Oncology Chatbots Through Large Language Models,” JMIR Bioinformatics and Biotechnology 5 (2024): e64406. 39321336 10.2196/64406 PMC11579624

