# Supplementary material for: Performance of Large Language Models in Answering Healthcare Delivery Questions: A Quantitative Cross‐Sectional Study
Source: Health Sci Rep. 2026 Jun 10;9(6):e72634. doi: 10.1002/hsr2.72634 (PMC13250468; doi:10.1002/hsr2.72634)
Supplement: Supplementary file 1 — Supporting File 1 [file HSR2-9-e72634-s001.docx]

Appendix 1. Study questionnaire.

| **No.** | **Question** | **A** | **B** | **C** | **D** |
| --- | --- | --- | --- | --- | --- |
| 1 | **Low-income Americans have less access to healthcare facilities and services compared to middle-income and high-income Americans. Which key concept from this lesson is highlighted by this statement?** | Epidemiology | Healthcare prevention | Public health | None |
| 2 | **The utilization of long-term care services is good for _____.** | Patients` family members only | Healthcare workers only | Patients only | None |
| 3 | **The patient of a primary care provider suffers a stroke and has to be placed in the intensive care unit at a certified stroke hospital. The hospital is an example of a _____ healthcare provider.** | primary | tertiary | secondary | None |
| 4 | **Which of the following statements about epidemiology is NOT true?** | Epidemiology helps us understand how diseases are distributed | Epidemiology helps us understand the causes of disease | Epidemiology mainly focuses on nutritional risk factors for disease | None |
| 5 | **What does it mean when a pharmacist tells a patient about a black box warning?** | That the side effect indicates a medical problem that is potentially contagious | That the side effect indicates a high risk of death or serious injury and to immediately seek medical help | That the side effect indicates a problem that the patient should report directly to the Food and Drug Administration | None |
| 6 | **Physicians that provide medical management for patients staying at a hospital facility are considered _____ providers.** | diagnostic | Rehabilitative | outpatient | None |
| 7 | **Which of the following is true regarding the patient's access to the pharmacist?** | The pharmacist is often the last health care professional to see the patient. | The pharmacist is often the first health care professional to see the patient. | The pharmacist has little ability to influence the patient's care. | None |
| 8 | **What is a responsibility of medical leaders?** | To serve as the spoke persons for medical staff | To build esteem of medical colleagues | To bridge the gap between staff physicians and the hospital board | None |
| 9 | **What is a disadvantage of evidence-based practice?** | the inability to find research on topics | the difficulty of finding valid credible evidence | discrediting of medical professionals' knowledge | None |
| 10 | **Who uses evidence-based practice?** | Only doctors and nurses | The patient family | The patient | None |
| 11 | **Which of these is a compilation of scientific research?** | randomized controlled trial | Case study | cohort study | None |
| 12 | **What is the CDC?** | Centers for Disease Control | Centers for Disease Control and Prevention | The local doctor's office | None |
| 13 | **What is required for emailing of patient health information?** | Encrypted email | Email must be sent on computer only | Include the patient date of birth and full name for identification | None |
| 14 | **What is information technology?** | The study of information | The use of information systems such as computers and databases to make information more accessible | The study of technology | None |
| 15 | The greatest benefit of adopting an electronic medical record system is _____. | Large storage capabilities | Reduction of paper waste | Accessibility of information | None |
